# Hepatitis B and C virus prevalence among patients and healthcare workers’ prevention practices towards the viruses in a secondary healthcare facility in Northern Nigeria

**DOI:** 10.11604/pamj.2023.46.82.40530

**Published:** 2023-11-13

**Authors:** Amaike Chikwendu, Harry Libby Unikutelle, Afolaranmi Tolulope Olumide

**Affiliations:** 1 Department of Community Medicine, Babcock University, Ilishan-Remo, Ogun State, Nigeria,; 2 Department of Community Medicine, Babcock University Teaching Hospital, Ilishan-Remo, Ogun State, Nigeria,; 3 Seventh-day Adventist Hospital, Jengre, Plateau State, Nigeria,; 4 Department of Community Medicine, University of Jos and Jos University Teaching Hospital, Plateau State, Nigeria

**Keywords:** Hepatitis B virus, hepatitis C virus, prevalence, occupational risk, practices, vaccination, healthcare workers, Nigeria

## Abstract

**Introduction:**

hepatitis B Virus (HBV) and hepatitis c virus (HCV) affect millions of people globally. Healthcare workers (HCWs) are at increased risk of infection due to occupation exposures where the viruses are spread mainly through needle stick injuries and exposure to infected blood and body fluid. The objective of this study was to assess the prevalence of viral hepatitis among patients and the practices of HCWs.

**Methods:**

this study involved a 5-years retrospective review of laboratory results of patients for HBV and HCV in addition to a questionnaire-based assessment of the preventive practices of 103 HCWs on HBV and HCV. Data was analyzed using SPSS.

**Results:**

the prevalence of HBV among the patients was 12.6% and 15.2% for HCV while the prevalence among the HCWs was 6.6% for HBV and 6.5% for HCV. About 60% of the HCWs had good overall preventive practices for viral hepatitis. Among the HCWs, 29.28% dropped needles in sharp containers after use, 53.5% recapped needles, 21.4% reused needles, 36.9% did not practice regular hand washing, and 53.4% completed the doses for HBV vaccine.

**Conclusion:**

there is high prevalence of HBV and HCV among the patients and the HCWs. This increases the occupational risk of infection with the viruses among HCWs. We recommend that more enlightenments and trainings be done for the HCWs to enable them take appropriate measures to protect themselves. Also, HCWs should provide HBV and HCV screening to patients accessing care and those found positive should be linked to care and treatment.

## Introduction

Viral hepatitis is an inflammation of the liver due to infection by the various hepatitis viruses, and it affects millions of persons globally. The subtypes of hepatitis virus include hepatitis A, B, C, D and E. However, only hepatitis B virus (HBV) and hepatitis C virus (HCV) result in chronic infections. About 296 million people and 58 million people are living with HBV and HCV respectively globally, leading to the death of about 1.1 million people yearly [1]. There is no vaccine to prevent Hepatitis C, however the virus can be controlled by treating those who are infected with antiviral agents which can cure more than 95% of those with the infection, while hepatitis B can be prevented by vaccination [1,2]. It is estimated that about 4.5 million premature deaths due to hepatitis B and C virus infections could be prevented in low- and middle-income countries by the year 2030 through measures such as vaccination, screening for hepatitis, use of drugs and health education [2]. In areas where HBV is endemic, it is most commonly spread by the perinatal or horizontal transmission. It is also spread by needlestick injury, tattooing, piercing and exposure to infected blood and body fluids. In addition, transmission may also occur through the reuse of contaminated needles and syringes or sharp objects either in health care settings, in the community or among persons who inject drugs [3]. HCV is also spread through similar routes as HBV; however, the perinatal route is less common. In healthcare settings transmission of the viruses occur mainly from the reuse or inadequate sterilization of medical equipment, especially syringes and needles, exposure to infected blood and body fluids and the transfusion of unscreened blood and blood products [3,4]. Provision of healthcare services exposes healthcare workers (HCWs) and their patients to increased risk of transmission of both HBV and HCV [5], primarily from unsafe injection practices, reuse of needles, fingerstick devices, and syringes, and other lapses in infection control in the healthcare settings. About 2 million HCWs are exposed and these exposures can result in about 70,000 cases of HBV and 15,000 HCV infections, with more than 90% of these infections occurring in developing countries [6]. Most of the blood exposures in healthcare settings are preventable. As part of measures to prevent the transmission of these viruses, HCWs should adhere to recommended standard precautions and fundamental infection-control principles. It is also recommended that HCWs with reasonably anticipated risk for exposures to blood or infectious body fluids should receive the complete doses of hepatitis B vaccine which consists of 3 doses given at an interval of 4 weeks [7]. Primary prevention interventions for HBV and HCV in healthcare settings include safe and appropriate use of health care injections, safe handling and disposal of sharps and waste, testing of donated blood for HBV and HCV before transfusion, and training of health personnel on preventive measures [4]. Other preventions strategies include increasing awareness through education of the public, vaccination against HBV, and early diagnosis and treatment [8]. Despite having good knowledge of HBV and HCV, the severity and the complications associated with the infections, some HCWs still have poor prevention practices [9]. A previous study was done in the Seventh-day Adventist (SDA) hospital Jengre, one of the SDA healthcare facilities in Northern Nigeria, to determine the prevalence of HCV among the clients who accessed care in the hospital from April 2017 to April 2018, which found a prevalence of 18.4% [10]. Study objectives: this study was aimed at assessing the prevalence of HBV and HCV infection among clients who accessed care in the hospital within the period reviewed and to assess the practices of HCWs on HBV and HCV in view of the high prevalence of HCV found in the earlier study.

## Methods

**Study design and study population**: the study was cross-sectional. It involved a five-year retrospective review of laboratory results of clients who assessed healthcare in SDA healthcare facilities from 2016 to 2020 and were screened for HBV and HCV, and a questionnaire-based assessment of the practices of the HCWs in SDA healthcare facilities on hepatitis B and C virus prevention. Study location: This study was done at the Seventh-day Adventist (SDA) healthcare facilities in northern Nigeria. This healthcare facilities are comprised of one secondary healthcare facility and eight primary healthcare facilities. The healthcare facilities provides both in-patient and out-patient care and they have equipped laboratories for investigations including HBV and HCV screening. Hepatitis B and C screening is recommended for ill patients on admission and at the General outpatient department (GOPD), pregnant mothers attending antenatal clinic, voluntary blood donors and as part of medical check-up for presumed healthy individuals. Rapid diagnostic test is done to determine the presence of hepatitis B surface antigen and HCV antibodies for HBV and HCV respectively.

**Eligibility criteria**: only clients who were screened for HBV and HCV and had complete information including results for HBV and HCV documented on the registers used for data extraction were included in this study. On the aspect of the HCWs, those who were absent during the time of data collection and those who did not give consent were excluded from the study.

**Study size for the healthcare workers**: appropriate sample size formula for cross-sectional study [11] was used to determine the minimum sample size for the HCWs, where n is the minimum sample size, Z_Þ_ is the value of alpha error at 95% confidence interval given as 1.96, p is the prevalence of HCWs that had overall good prevention practices of 42.6% from a study in southwest Ethiopia, [9], q is 1-p, and d is the precision set at 10%. In order to account for non, poor or incomplete responses, 10% of the calculated sample size was added given a minimum sample size of 103.

**Selection of participants**: simple random sampling by balloting was used to select the study participants among the HCWs.

**Collection of data**: the laboratory request registers and laboratory result registers were used to collect data. The request register contained information on the age of the patient, address, sex, the category of the patients and the various investigations the patient was requested to do, while the result register contained the results of the investigation done in addition to the other information on the request register. Both registers were used to ensure quality of data collected as necessary information missing from one register was found in the other register. A data extraction form was used for data collection. The form contained information on age, address, sex, category of patient in terms of the reason for the investigation, HBV result and HCV result. The laboratory data collection was done manually over a period of 6 months. Semi-structured questionnaire adapted form studies in the Ethiopia, Democratic Republic of Congo (DRC) and Vietnam [9,12,13] was used to obtain information from the HCWs. The questionnaire had two sections (sociodemographic characteristics and practices) Five research assistants (1 doctor, 2 laboratory technicians, and 2 health records officers) who were involved in data collection were trained on the content of the questionnaire and methods of administration and methods for data extraction from the laboratory registers. Questionnaire was pre-tested among similar HCWs in a different healthcare facility for clarity and correction of any ambiguity. Data collection was by self-administered questionnaire in the presence of the research assistants to avoid exchange of information among the HCWs or from getting answers from the internet. Study participants were reassured of confidentiality for information they provided.

**Data processing and analysis**: data was processed and analyzed using SPSS version 23, manufactured by International Business Machines Corporation, Armonk, New York, United States of America. Age for the clients was graded as < 18 and ?18 years, while for the HCWs it was grouped as 16-25, 26-35, 36-45, 46-55 and 55-65 years. Sex was assessed as male and female based of the responses of the HCWs and the clients. Category of clients was done based on the purpose of presentation in the hospital, and the categories include ANC attendee for those attending ANC, blood donors for voluntary blood donors, ill-patient for patients who presented on account of ill-health and medical check-up for apparently healthy clients who came for medical check-up. Quantitative variable like practice score was summarized using mean and standard deviation. Preventive Practices for HBV and HCV was accessed jointly. A maximum of 21 score and a minimum of 6 scores were attainable. Any score less than 14 was considered poor practice while any score of 14 and above was considered as good practice. Explanatory variables like age group, sex, educational status, marital status, years of working experience, cadre, and category of client were presented on frequency tables as frequency and percentages. Prevalence was reported as percentage.

**Ethical consideration**: written permission was sought and obtained from the Institutional Review Board of the SDA Hospital Jengre. This study was conducted in accordance with the guidelines and regulations of the approving institution. Written permission was also gotten from the head of the laboratory unit to access the registers and data from the laboratory. In addition, written informed consent was obtained from all the HCWs who participated in the study. They were also reassured of confidentiality and anonymity for the information they will provide and this was maintained. Clients who access care in SDA Hospital Jengre are counselled and requested to give anticipatory consent for future research. In order to ensure confidentiality, documents used for data collection were stored in a locked file cabinet and personal identifiers removed from study documents as soon as data collection was completed.

## Results

A total of 7071 results of clients who had HBV and HCV screening was reviewed out of which 230 had some missing information. Also, a total of 103 HCWs were studied for their preventive practices on HBV and HCV. Majority 6427 (94.2%) of the clients were 18 years and above. There were more males 4129 (60.4%) compared to females 2712 (39.6%). Clients who were screened for HBV and HCV on account of ill-health were 4536 (66.3%), (Table 1, Table 2). A total 861 of the clients were positive for HBV giving a prevalence for the period studied, of 12.6% (95% CI: 11.8 to 13.4%), while 1037 were positive for HCV giving a prevalence of 15.2% (95% CI: 14.4 to 16.1%) and 108 had coinfection of HBV and HCV with a prevalence of 1.6%. Except for the year 2017, the number of clients screened had a progressive increase so as the number of those positive for HBV and HCV (Table 1, Table 2).

**Table 1:** sociodemographic characteristics of the clients and prevalence of HBV and HCV

	Frequency	Percentage (%)
**Variable**		
**Age (years)**		
<18	399	5.8
≥ 18	6427	94.2
**Sex**		
Male	4129	60.4
Female	2712	39.6
**Category of clients**		
ANC attendee	576	8.4
Blood donor	1564	22.9
Ill-patient	4536	66.3
Medical check up	165	2.4

**Table 2:** prevalence of viral hepatitis from 2016 to 2020

Year of Study	2016	2017	2018	2019	2020	Total (Prevalence)
**Number of clients tested**	1133	991	1142	1458	2117	**6841**
**HBV positive**	134	120	150	182	275	**861 (12.6%)**
**HCV positive**	221	125	134	238	319	**1037 (15.2%)**
**HBV/HCV positive**	23	7	14	23	41	**108 (1.6%)**

There are slightly more females 55 (53.4%) in the study compared to the males 48 (46.6%) HCWs, with 58(56.3%) of the respondents having less than 6 years of working experience. The age group 26-35 years had the highest number of participants, 35 (34%), and 31 (30.1%) of the respondents were Community health workers, Table 3. The mean preventive practice score was 15.89 ± 3.26 and 62 (60.2%) of the respondents had good overall hepatitis virus prevention practices. Ninety-one (90.1%) of the respondents have ever been screened for HBV while 77(74.8%) for HCV, with 6(6.6%; 95% CI: 1.8 to 12.5%) positive for HBV, while 5(6.5%; 95% CI: 2.1to 14.5%) were positive for HCV. More than half {56 (54.9%)} knew about the existence of a post-exposure prophylaxis (PEP) or Infection Prevention and Control (IPC) committees in the hospital. Only 55 (53.4%) have completed the 3 doses of HBV vaccine. Twenty-six (25.2%) have been exposed to patients´ body fluid or had needle prick in the last 6 months prior to this study. Out of 89 of the HCWs who use needles for procedure, 63 (70.8%) dropped needle in sharp containers after use, 46 (51.7%) recapped needles, and 19 (21.3%) reused needles. About 63% of the HCWs practiced regular hand washing, Table 4.

**Table 3:** sociodemographic characteristics of the healthcare workers

	Frequency	Percentage (%)
**Variable**		
**Age**		
16-25	23	22.3
26-35	35	34.0
36-45	22	21.3
46-55	15	14.6
56-65	8	7.8
**Sex**		
Female	55	53.4
Male	48	46.6
**Marital status**		
Never married	50	48.5
Widowed	7	6.8
Married or living together	46	44.7
**Level of Education**		
Primary	7	6.8
Secondary	35	34.0
Tertiary	61	59.2
**Category of HCW**		
Pharmacists/Pharmacist technicians	7	6.8
Laboratory	10	9.7
Doctors	5	4.9
Nurses/Midwives	9	8.7
Community health workers	31	30.1
Ward attendants	12	11.6
Students on clinical placements	15	14.6
Laundry staff	3	2.9
House keepers	7	6.8
Administrative staff	4	3.9
**Years of working experience**		
< 6	58	56.3
6-10	13	12.6
11-15	10	9.7
16-20	6	5.8
≥ 20	16	15.5

**Table 4:** prevention practices on viral hepatitis infection

Variable	Frequency	Percentage
**Ever screened and know HBV status**	91	88.3
**Known positive HBV status**	6	6.6
**Screened for HBV in the past 6 months**	41	39.8
**Ever screened and know HCV status**	77	74.8
**Known positive HCV Status**	5	6.5
**Screened for HCV in the past 6 months**	31	30.1
**Treated for HCV**	1	20
**Ever received at least a dose of HBV vaccine**	77	74.8
**Completed the dose of HBV vaccine**	55	53.4
**Reason for no vaccination**		
Don’t know where to get the vaccine	3	11.5
Not at risk	6	23.1
Not aware of HBV vaccine	7	27.0
Not in support of vaccination	1	3.8
Vaccine not effective	3	11.5
Afraid of taking injections	4	15.4
The vaccine is expensive	2	7.7
**Reason for not completing dose of HBV vaccine**		
Busy schedule	4	16
Forgot appointment date	11	44
Long waiting time between doses	4	16
Thought I was protected from the doses taken	6	24
**Exposed to patients’ blood or needle prick in the past 6months**	26	25.2
**Action taken following exposure**		
Washed hand with soap and water	14	53.8
Reported to IPC committee	8	30.8
Reported to doctor	11	42.3
Did nothing	1	3.8
Screened for HBV/HCV	10	38.5
Screened for HIV	7	26.9
Took traditional medicine	2	7.7
**Aware of HBV vaccine in this hospital**	72	69.9
**Awareness of PEP or IPC committee in the hospital**	56	54.4
**Use of gloves for procedures/waste disposal**	90	87.4
**If yes to use of gloves, how often**		
Always	47	52.2
Sometimes	43	47.7
**Drop needle in sharp container after use**	63	**70.8**
**Recap needles after use**	46	**51.7**
**Reuse needles**	19	**21.3**
**Frequency of hand hygiene while attending to patients**		
Always	65	63.1
Sometimes	38	36.9
**Overall Preventive Practice Level**		
Good	62	60.2
Poor	41	39.8
**Mean Preventive practice score = 15.89 ± 3.26**		

## Discussion

Hepatitis B and C virus infection are diseases of serious public health importance considering the burden of morbidity and mortality associated with them [2]. This study assessed the prevalence of HBV and HCV among clients who accessed care at SDA hospital Jengre, and the preventive practices of the HCWs in SDA Hospital on HBV and HCV. Males had most of the laboratory results, with majority of the clients aged 18 years and above, while about two-thirds of the clients screened for HBV and HCV were screened on account of ill health. In this study the prevalence of HBV was 12.6% and HCV was 15.2% while multimorbidity of HBV and HCV was 1.6%. These findings are higher than the global prevalence of HBV and HCV and the area where this study was conducted is of high prevalence based on WHO epidemiological classification of HBV and HCV prevalence [3,4,14]. The findings are also higher than the prevalence of HBV of 8.1% and HCV of 1.1% in a nationwide wide survey in Nigeria [15], and 12.2% for HBV in another Nigeria national survey [16]. Furthermore, the prevalence of HBV in this study was also higher than the pooled prevalence of 9.5% for HBV in another study in Nigeria [17]. Also, the prevalence of HCV was higher than the findings 6.9% from a similar study in Northeast Nigeria [18], and 4.3% in Northwest Ethiopia [19]. Again, the HBV/HCV co-infection in this study was higher than findings from a study in Northwest Ethiopia of 0.9% [19].

The high prevalence of HBV and HCV in this study may be due the fact that this study was done in a rural setting where most of the participants may not have good awareness of the viruses and also the various methods of prevention for the viruses. It may also be due to some sociocultural practices like multiple sexual partners, polygamous marriages and scarification marks. In addition, considering the high prevalence of HBV and HCV, all the adults in this environment where the study was conducted should have access to and be offered HBV and HCV testing with linkages to prevention, care and treatment services based on WHO recommendations [3,4]. The prevalence of HBV among the healthcare workers was 6.6% and 6.5% for HCV. This may be a reflection of the high prevalence found among the patient who access care in the healthcare facility were this study was conducted. This finding was higher than that of a study in Southwest Ethiopia of 2.5% and 0.42% for HBV and HCV respectively [9] and in Tunisia 0.13% for HCV but it is lower than the prevalence of HBV of 10.6% among HCWs in Cameroon. The high prevalence is Cameroon was attributed to the high prevalence in the general population and also low level of knowledge of the routes of transmission among the HCWs [20]. Most of the healthcare workers had good overall preventive practices for HBV and HCV. This finding was higher than the 42.6% good practice in the study in Southern Ethiopia [9]. This may be due to the high prevalence of viral hepatitis in this study setting making the HCWs to adopt good prevention practices. The finding in this study was however lower than that of 65.5% good preventive practices among HCWs in Sudan. This may be due to the fact that the study in Sudan was among more skilled HCWs only while our study involved both skilled and unskilled HCWs.

Majority of the HCWs have been screened for both HBV and HCV. The number of HCWs in this study screened for HBV is higher when compared to the finding of 71.4% of HCWs in a study done in Northern Uganda. Screening and knowing ones HBV and HCV status is vital in the prevention as those who test negative would receive the HBV vaccine and be protected from been infected while those who test positive will commence treatment early. These measures will also prevent HCWs from transmitting the viruses to their clients [21]. About three-quarters of the respondents have receives at least one dose of HBV and out of this about half of them completed the 3 doses required for HBV prevention. This finding was higher than that of a study in Democratic Republic of Congo (DRC) where 24.4% of the HCWs have ever received HBV vaccine out of which only 3.8% completed the 3 doses. The low rate of vaccination in DRC was attributed probably to the disorganization of the health system following the sociopolitical instabilities in the country as at the time of their study [12]. It is also higher than the 23.4% who received at least one dose in a study in Cameroon and this was attributed to the poor knowledge of HBV among the HCWs [20]. The percentage of HCWs who completed the three doses of HBV in this study was also higher than that of 40.9% found in Sudan [22]. Again, following accidental exposure to the body fluid of patients as also found in some other studies in DRC [12] and Northern Uganda [21], some of the respondents were more concerned about screening for HIV instead HBV and HCV. This may be due to the poor awareness on hepatitis as compared to HIV since HIV has well organized programs and treatment clinics in most healthcare facilities. There is need for more awareness to be created among HCWs on the high infectivity of HBV and HCV when compared to HIV [12]. The high prevalence of HBV and HCV among the clients in this study also increases the occupational risk of the HCWs of getting infected with the viruses since close to half of the HCWs have not received the complete doses of HBV vaccine. Also, some of the healthcare workers do not adhere to recommended standard precautions and fundamental infection control principles, as some still do not wash their hands before and after procedures, do not use gloves always, still reuse and recap needles, do not properly dispose of used needles and sharps. Some of these findings were also reported in studies among HCWs in Cameroon [20], and in Northern Vietnam [13] These practices increase the occupational risk of infection with HBV and HCV. There is need for more enlightenment and training for the HCWs to observe universally accepted precautions in providing care for clients in order to reduce the risk of getting infected with viruses and transmitting same to other clients [1]. This study combined secondary data from clients who accessed care in the hospital and primary data from HCWs in the hospital. This is a strength for this study.

**Limitations of study**: The retrospective nature using secondary data collection may have been a limitation to this study as some data were missing from the laboratory registers reviewed. However more than one registers were used to reduce the number of missing data. Also, HBV and HCV were only tested using rapid test kits. Additional tests to determine the presence of Hepatitis B envelope antigen (HBeAg) and HCV ribonucleic acid (RNA) test should have been done to determine active infection among those who tested positive to HBV and HCV respectively. In addition, information gotten from the HCWs was by self-reporting and this is subject to individual bias. In order to reduce this, the respondents were assured of their anonymity and confidentiality of information they provided.

## Conclusion

This study showed a high prevalence of HBV and HCV among patients and HCWs. Even though more than half of the healthcare workers had good overall preventive practices for viral hepatitis, some of the healthcare workers did not adhere to universal preventive precautions in their practices. We recommend that more awareness be created for HCWs to adhere to universal best practices in order to prevent themselves from been infected with viral hepatitis and also recommend that the HBV and HCV should be recommended routinely for patients accessing care in the healthcare facility and those who are positive should be linked to care and treatment while those who are negative should be encouraged to receive the full dose of HBV vaccine.

### What is known about this topic

- *Most study in this area is either on HBV or HCV;*
- *Prevalence of HBV and HCV is high in the WHO African region*.

### What this study adds

- *This study combined the review of past records of patients and primary information of HCWs;*
- *This study assessed the prevalence of HBV and HCV among the patients and the HCWs*.
